# Exposure to TiO_2_ nanoparticles increases *Staphylococcus aureus* infection of HeLa cells

**DOI:** 10.1186/s12951-016-0184-y

**Published:** 2016-04-22

**Authors:** Yan Xu, Ming-Tzo Wei, H. Daniel Ou-Yang, Stephen G. Walker, Hong Zhan Wang, Chris R. Gordon, Shoshana Guterman, Emma Zawacki, Eliana Applebaum, Peter R. Brink, Miriam Rafailovich, Tatsiana Mironava

**Affiliations:** Department of Materials Science and Engineering, Stony Brook University, Stony Brook, NY USA; Department of Bioengineering, Lehigh University, Bethlehem, PA USA; Department of Oral Biology and Pathology, School of Dental Medicine, Stony Brook University, Stony Brook, NY USA; Department of Physiology and Biophysics, Stony Brook University, Stony Brook, NY USA; Yeshiva University High School for Girls, Hollis, NY USA; University of California at Los Angeles, Los Angeles, CA USA; Stern College for Women, New York, NY USA

**Keywords:** Titanium dioxide, Nanoparticles, Nanotoxicology, HeLa cells, Bacterial infection

## Abstract

**Background:**

Titanium dioxide (TiO_2_) is one of the most common nanoparticles found in industry ranging from food additives to energy generation. Approximately four million tons of TiO_2_ particles are produced worldwide each year with approximately 3000 tons being produced in nanoparticulate form, hence exposure to these particles is almost certain.

**Results:**

Even though TiO_2_ is also used as an anti-bacterial agent in combination with UV, we have found that, in the absence of UV, exposure of HeLa cells to TiO_2_ nanoparticles significantly increased their risk of bacterial invasion. HeLa cells cultured with 0.1 mg/ml rutile and anatase TiO_2_ nanoparticles for 24 h prior to exposure to bacteria had 350 and 250 % respectively more bacteria per cell. The increase was attributed to bacterial polysaccharides absorption on TiO_2_ NPs, increased extracellular LDH, and changes in the mechanical response of the cell membrane. On the other hand, macrophages exposed to TiO_2_ particles ingested 40 % fewer bacteria, further increasing the risk of infection.

**Conclusions:**

In combination, these two factors raise serious concerns regarding the impact of exposure to TiO_2_ nanoparticles on the ability of organisms to resist bacterial infection.

**Electronic supplementary material:**

The online version of this article (doi:10.1186/s12951-016-0184-y) contains supplementary material, which is available to authorized users.

## Background

Titanium dioxide (TiO_2_) is naturally occurring compound that has several polymorphs with the same chemical formula but different crystalline structures. Rutile and anatase are the most abundant forms of TiO_2_, both of which have tetragonal crystal structure, and only differ in atomic arrangement [1]. TiO_2_ is generally used as a white pigment due to its brightness and high refractive index and accounts for 70 % of the total production volume of pigments worldwide [2]. Roughly four million tons of TiO_2_ (nano and bulk combined) are used for annual production of paints, coatings, plastics, inks, paper, pharmaceuticals, cosmetics, toothpastes, medicines, sunscreens and food products [3–7]. Annual production of nanosized TiO_2_ was estimated to reach 200,000 metric tons in 2015 [8] bringing TiO_2_ NPs to the top five nanoparticles (NPs) used in consumer products [9].

These particles also exhibit photocatalytic activity and have been intensively studied in anti-cancer and anti-bacterial applications. The first study of anti-cancerous activity of TiO_2_ nanoparticles in human cervix adenocarcinoma (HeLa) model was performed in the early 1990s by Cai et al. [10, 11] who showed that HeLa cells could be effectively destroyed by TiO_2_ upon short irradiation with UV light. Within a decade, the effectiveness of TiO_2_ in combination with UV light as an anti-cancerous agent, was confirmed by multiple groups in different cancer models [12–24]. The efficacy of TiO_2_ and UV light against Gram-negative and Gram-positive bacteria [25–27] was reported even earlier, and by now is a well-established phenomenon [28].

Recently, several research groups reported that TiO_2_ NPs exhibit immediate toxicity and also can induce genotoxicity [9, 29–31] in ambient light and dark conditions without exposure to UV light. In fact, the International Agency for Research on Cancer has recently classified the TiO_2_ particles as “a possibly carcinogenic to humans”. The detrimental effect of these particles is well understood in terms of reactive ion species formed, which are toxic to both eukaryotic cells and bacteria, when the photoelectron is emitted after irradiation of TiO_2_ particles. Yet, in the absence of UV irradiation, TiO_2_ is reported to be toxic primarily to eukaryotic cells and not to bacteria [32–34]. This can be a cause of possible concern, especially when the cells exposed to particles are also exposed to bacteria. Hence in this paper we chose to focus on this situation, where TiO_2_ particles in conjunction with radiation, have been previously studied separately in two systems.

The bacterial system we chose is *Staphylococcus aureus (S. aureus)* which is one of the most successful human pathogens with very diverse range of virulence factors and is the leading cause of human infections worldwide [35–39]. The bacteria resides in the anterior nares of 20–30 % of humans [40, 41] and, besides being resistant to numerous antibiotics, is also able to evade host immune system [42–44]. Consequently, as reported by Gaupp el al. [45] it is capable of causing an array of diseases from minor soft tissue infections to life-threatening septicemia. Previous work had shown that these bacteria were highly susceptible to ROS products and exhibited a well-defined exclusion zone when exposed to high concentrations of TiO_2_ [46, 47]. Since these concentrations are also toxic to cells, we chose to focus on the effects at low concentrations, where ROS production is negligible and which were previously shown not to affect cell proliferation, yet as we will demonstrate, can still have profound effects on cell function and the interaction of cells with bacteria.

## Results

**Fig. 1:**
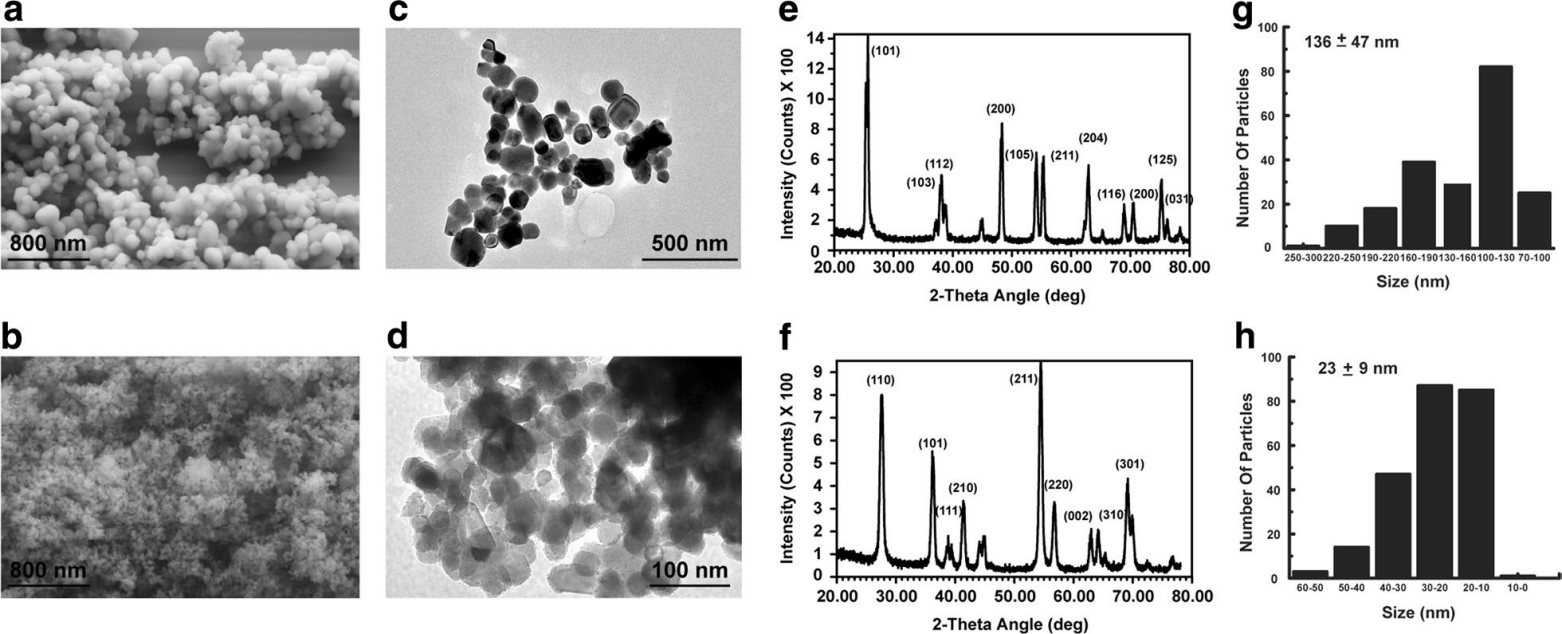
TiO_2_ nanoparticles imaged by TEM and SEM, their size distribution histograms and X-ray diffraction spectra. SEM picture of anatase (**a**) and rutile (**b**) TiO_2_ nanoparticles; TEM picture of anatase (**c**) and rutile (**d**) TiO_2_ nanopartiles; X-ray diffraction spectra of anatase (**e**) and rutile (**f**); size distribution histograms of anatase (**g**) and rutile (**h**)

The TEM and SEM images of rutile and anatase TiO_2_ are shown in Fig. 1, together with a histogram of the particle size distribution. From the figure we see that both rutile and anatase particles have a spherical shape, with anatase particles being significantly larger than rutile. From TEM images, the calculated average diameter of rutile is 23 ± 9 nm and the average diameter of anatase is 136 ± 47 nm. X-ray diffraction spectra of both particles are shown on Fig. 1e, f confirming anatase and rutile crystal structures. The surface charges of the particles in deionized water were measured using zeta potentiometry, and found to be −34.75 ± 1.63 and −26.94 ± 0.56 mV for anatase and rutile respectively. But after incubation in DMEM for at least 24 h their zeta potentials were found to −7.39 ± 0.90 and −7.35 ± 0.73 mV for anatase and rutile respectively. Particle aggregation in complete medium was accessed by DLS measurement. The average NPs sizes were 355 ± 37 and 73 ± 1 nm for anatase and rutile respectively, indicating particle aggregation. The average aggregates consist of three nanoparticles for both anatase and rutile. Such small aggregation may only insignificantly influence the nanoparticle–cell interaction. It was previously shown that effects dependent on the particle’s free surface (such as free radical production) diminish as particles aggregate. On the other hand, phagocytosis appears to be more efficient for aggregates than for single particles counterbalancing effect of decreased surface area [48].

**Fig. 2:**
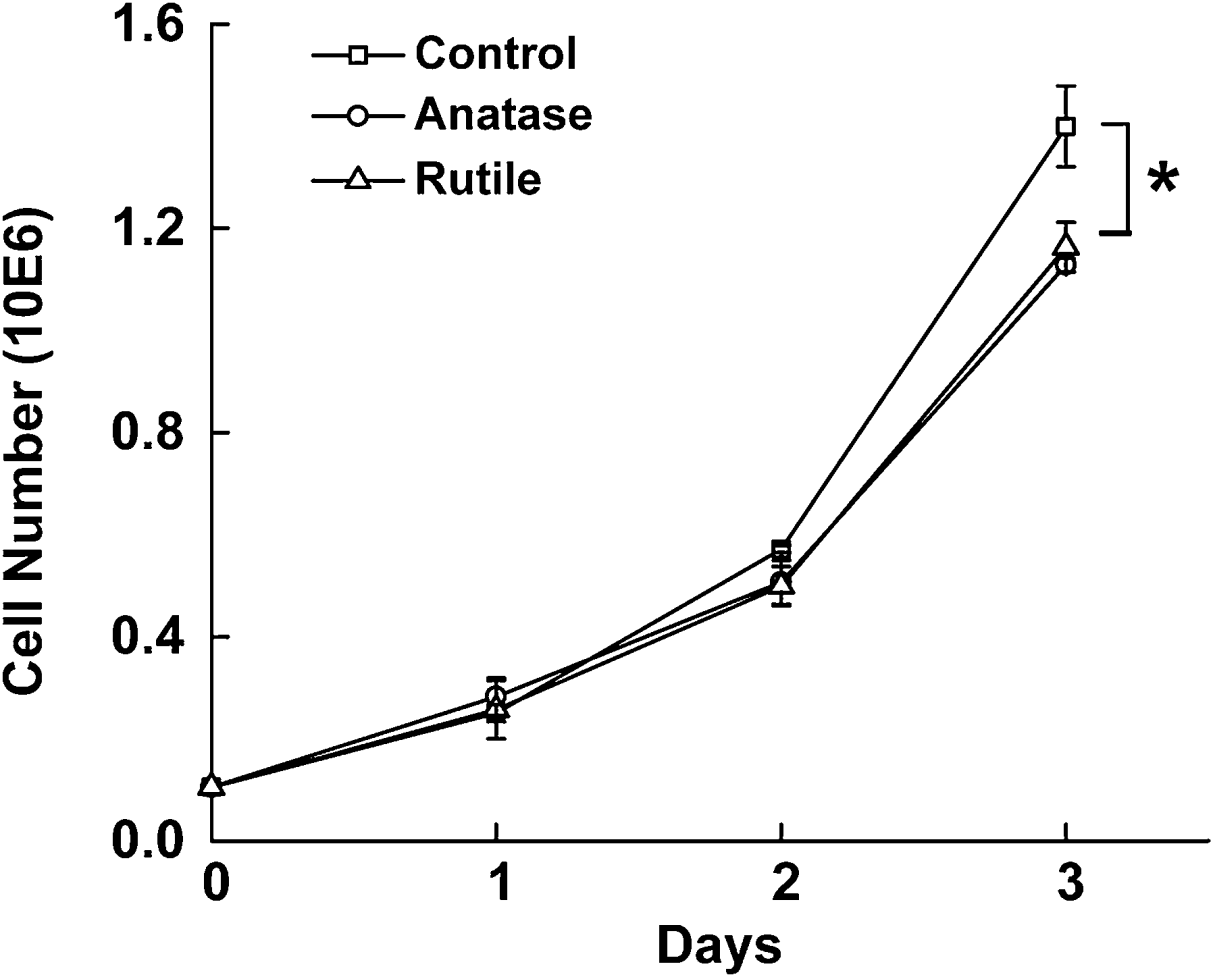
Proliferation of HeLa cells exposed to 0.1 mg/ml anatase and rutile TiO_2_ for 3 days and control unexposed cells

In order to determine TiO_2_ NPs’ toxicity at 0.1 mg/ml concentration and to avoid false reading in MTT assay induced by formazan precipitation from TiO_2_-MTT reaction [49], we measured cell proliferation using standard cell counting. From Fig. 2a we can see that cell cultures treated with 0.1 mg/ml of TiO_2_ for 24 and 48 h did not exhibit any changes in cell proliferation compared to control. Only after 72 h of exposure, a decrease in cell proliferation was observed, however it did not exceed 16 % for both rutile and anatase. Since the proliferation rate of cell population may be reduced if the length of the cell cycle increases due to the changes in metabolic activity we also monitored the cell population doubling times. We didn’t detect any changes in cell doubling times during first 2 days of exposure to TiO_2_ NPs, on day 3 slight changes in the cell doubling times was detected in the cultures exposed to TiO_2_ NPs confirming the proliferation data (Additional file 1: Figure S1).

**Fig. 3:**
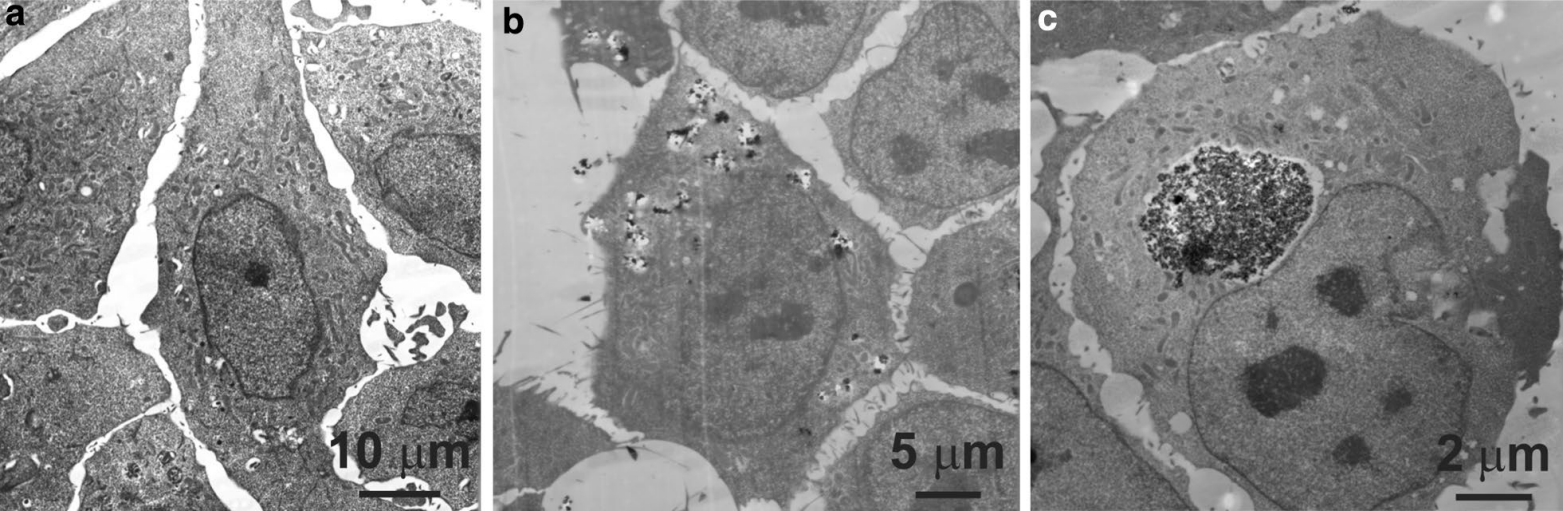
TEM cross section of HeLa control cells (**a**), cells exposed to 0.1 mg/ml anatase (**b**) and 0.1 mg/ml rutile (**c**)

Electron micrograph images show that either particles are sequestered in vesicles within cells or in the process of being endocytosed following a 24-h exposure to TiO_2_. TEM cross sections of HeLa cells exposed to rutile and anatase particles are shown in Fig. 3, from which we can see that rutile (Fig. 3c) particles are typically stored in a few very large vacuoles (average size of 6.22 µm) occupying roughly 25–35 % of the cell cross-section. Alternatively Fig. 3b reveals that anatase nanoparticles are stored in multiple smaller vacuoles (~0.864 µm) which are distributed across the cell. In both cases no evidence of particles penetrating either nuclei or mitochondria was found.

Internalization of TiO_2_ nanoparticles by HeLa cells was also confirmed by flow cytometry where natural florescence of these particles was gated in separate channels. In Table 1 we can see that in fluorescent channel FL4-H most of control cell population is gated with M1 marker (autofluorescence) and only 0.37 % of population is gated with M2 marker (TiO_2_ fluorescence). After exposure to TiO_2_ nanopartiles for 24 h cell population gated with M2 marker increased up to 1.11 and 9.62 % for rutile and anatase respectively indicating uptake of nanoparticles (Additional file 1: Figure S3).

**Table 1:** Percentage of HeLa cells with auto- and TiO_2_—induced fluorescence

Sample	Fluorescence, M2 (TiO_2_ induced) (%)	Fluorescence, M1 (auto) (%)
HeLa control	0.37	97.68
HeLa + TiO_2_ anatase	9.62	83.47
HeLa + TiO_2_ rutile	1.11	92.48

**Fig. 4:**
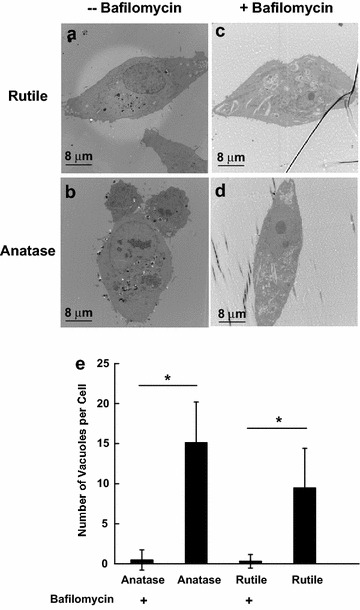
TEM cross sections of HeLa cells exposed to **a** 0.1 mg/ml rutile and **b** 0.1 mg/ml anatase, TEM cross sections of HeLa cells treated with bafilomycin prior to exposure to **c** 0.1 mg/ml rutile, and **d** 0.1 mg/ml anatase; **e** number of vacuoles per HeLa cell exposed TiO_2_ with and without bafilomycin pre-treatment. *Means P < 0.05

When particles were added to cell cultures as suspension, it is important to know how TiO_2_ NPs enter cells. To investigate particle penetration we used bafilomycin to block vacuolar ATPases and inhibit endocytic activities. As shown in Fig. 4, the uptake of TiO_2_ nanopaticles by HeLa cell is almost completely inhibited in the culture treated with bafilomycin. On the other hand, cultures not exposed to bafilomycin have on average nine and 16 vacuoles filled with TiO_2_ NPs per cell for rutile and anatase treatments respectively.

**Fig. 5:**
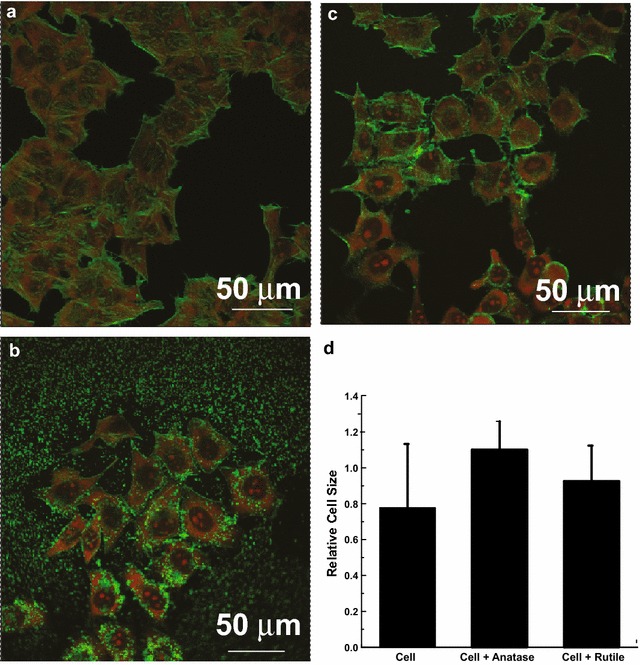
HeLa cells imaged with confocal microscopy after 24 h in culture: **a** Control; **b** cells exposed to 0.1 mg/ml anatase, and (**c**) 0.1 mg/ml rutile. The actin cytoskeleton of HeLa cells was visualized using the green-fluorescent Alexa Fluor^®^ 488 phalloidin and the nucleus was stained with propidium iodide. **d** Average size of HeLa cells exposed to 0.1 mg/ml TiO_2_ NPs for 24 h and unexposed control

To determine if abnormal changes occur to cell morphology due to nanoparticles exposure we performed a confocal microscopy study. The images obtained for cells incubated with two types of TiO_2_ after 24 h of exposure are shown on Fig. 5a–c, from which it can be seen that HeLa cells not exposed to nanoparticles are adherent to each other and occupy approximately the same area. After exposure to rutile and anatase nanoparticles, morphological signs of damage become apparent as cells failed to establish connections between each other and become isolated. From the Fig. 5d we can see that no change in the cell size was observed upon exposure to TiO_2_ NPs. In order to determine if increased ROS is responsible for HeLa cells failure to establish connections, we measured ROS generated in the cultures that exposed to rutile and anatase for 24 h. As shown in Additional file 1: Figure S2, even though a small increase in ROS is observed its magnitude is not statistically significant (p > 0.50).

**Fig. 6:**
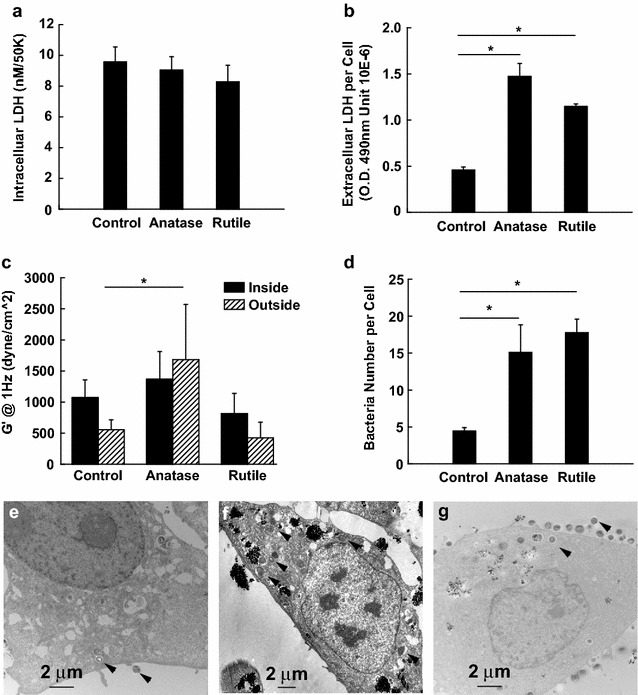
Lactate dehydrogenase amount in HeLa control cells and cells exposed to 0.1 mg/ml anatase, and 0.1 mg/ml rutile TiO_2_. **a** Intracellular LDH; **b** Extracellular LDH. **c** Microrheology of HeLa cells exposed to TiO_2_. **d** Number of *S. aureus* bacteria per HeLa cell exposed to anatase, rutile and unexposed to TiO_2_ nanoparticles. TEM cross sections of HeLa control cells (**e**) and cells exposed to 0.1 mg/ml anatase (**f**), and 0.1 mg/ml rutile TiO_2_ (**g**) followed by exposure to *S. aureus* bacteria for 90 min. *Arrows* point to bacteria. *Means P < 0.05

While cell proliferation provides information about cytocompatibility of NPs, secondary processes triggered by exposure to TiO_2_ NPs may determine the long-term toxicity effects. In this regard, the release of Lactate-dehydrogenase (LDH), which has been found to be associated with the loss of cell-membrane integrity, is another indicator of cellular toxicity induced by NPs. Assay for extracellular LDH in HeLa cells pretreated with TiO_2_ NPs (Fig. 6b) revealed threefold and twofold increase in extracellular LDH levels in the cultures treated with anatase and rutile, respectively. To ensure that observed increase in extracellular LDH results from the loss of membrane integrity and not from increased LDH secretion (possibly stimulated by exposure to TiO_2_ NPs) we also tested amount of LDH inside of the cell (intracellular LDH). From the Fig. 6a we can see only insignificant change in the intracellular LDH levels in HeLa cells treated with rutile or anatase. The increased cytotoxicity observed in HeLa cells treated with anatse could be attributed to enhanced uptake of anatase that was previously indicated by the flow cytometry.

Effects of nanoparticle exposure on intracellular and membrane rheology were assessed by optical tweezers. Results on Fig. 6c indicate that intracellular rheology is not affected by exposure to nanoparticles for 24 h. On the other hand, HeLa cell membrane became significantly harder (<150 %) after exposure to anatase nanoparticles, while exposure to rutile had no effect on the membrane stiffness.

**Fig. 7:**
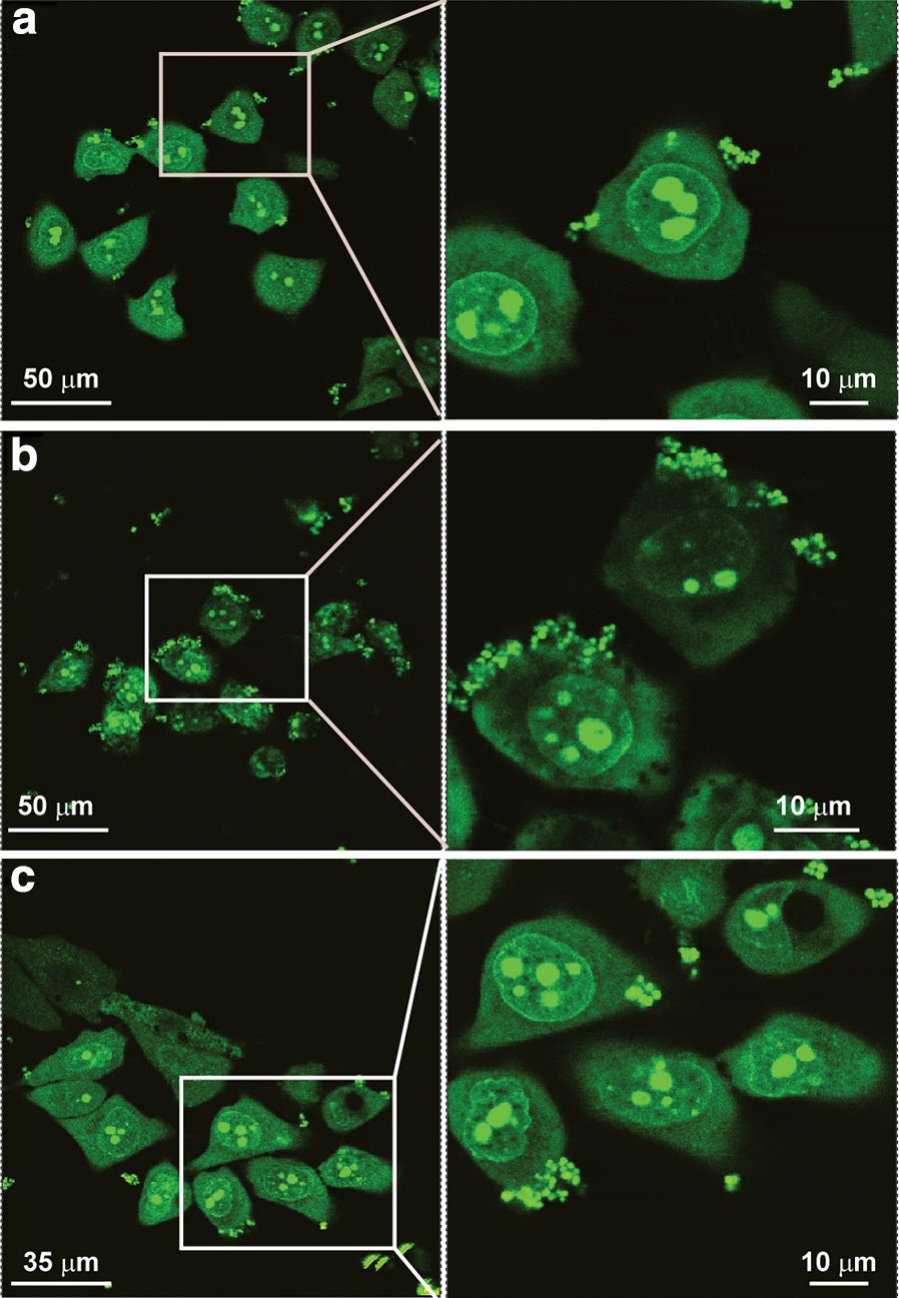
Confocal microscopy pictures of HeLa control cells (**a**) and cells exposed to 0.1 mg/ml anatase (**b**), and rutile TiO_2_ (**c**) followed by exposure to *S. aureus* bacteria. The cells and bacteria were both stained* green* by SYTO 9. *Arrows* point to bacteria (*green dots*)

After we observed changes in cellular membrane after exposure to TiO_2_ nanopartilces, we decided to investigate if that change affects cellular resistance to bacterial infections. HeLa cells treated with TiO_2_ NPs for 24 h, prior to *S. aureus* exposure, internalized and bound more *S. aureus* cells than control HeLa cells that were not treated with NPs prior to bacterial exposure. Figure 6g shows a representative HeLa cell that had been pretreated with NPs prior to exposure to *S. aureus*. Note the internalized and plasma membrane-associated *S. aureus*. Based on colony formation units (CFU), HeLa cells treated with anatase and rutile NPs had more *S. aureus* bound/internalized per HeLa cell than control HeLa cells that were exposed to *S. aureus* but were not pretreated with NPs (Fig. 6d). CFU counts determined that HeLa cells treated with anatase had 2.5-fold more bacteria per HeLa cell than control HeLa cells. Rutile treated HeLa cells had approximately 3.5-fold more *S. aureus* per HeLa cell, based on CFU’s, then control cell that were not treated with anatase. Confocal microscopy (Fig. 7) agreed with the CFU data and demonstrated that HeLa cells treated with TiO_2_ NPs prior to exposure to *S. aureus* had more bacteria internalized and bound to the HeLa cell membrane than control cells that had not been pretreated with NPs. We are confident that the *S. aureus* cells were tightly bound to the HeLa plasma membrane, as shown in Figs. 6g and 7, since vigorous washing of the tissue culture plate three times with PBS did not remove the *S. aureus*. Therefore, the data presented in Figs. 6 and 7 show that preexposure to TiO_2_ NPs resulted in increased internalization of *S. aureus* and that more *S. aureus* cells became tightly bound onto the plasma membrane of the HeLa cells compared to control cells.

**Fig. 8:**
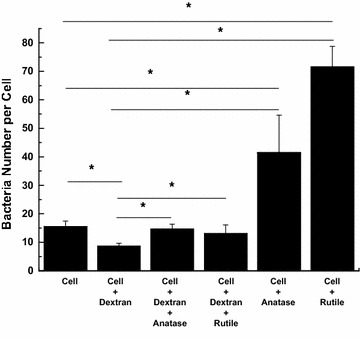
Number of *S. aureus* bacteria per HeLa cell pretreated with Dextran (0.1 mg/ml) for 24 h and followed by exposure to 0.1 mg/ml anatase and rutile TiO_2_ NPs

To understand the mechanism of increased bacteria uptake in cells exposed to TiO_2_ NPs we inhibited bacteria adhesion to the cell membrane by exposing HeLa cells to dextran prior to introduction of *S. aureus* bacteria. Our results indicate (Fig. 8) that in control cultures unexposed to TiO_2_ NPs dextran inhibits bacteria uptake by twofolds, however in cultures exposed to rutile and anatase TiO_2_ NPs pre-treatment with dextran results in decrease in bacteria uptake by 3.5 and fivefolds respectively.

**Fig. 9:**
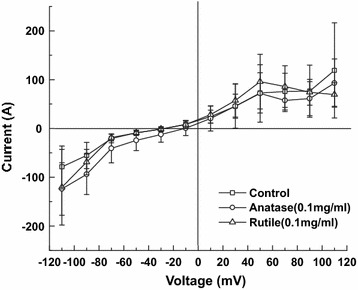
Steady state currents of HeLa control cells and cells exposed to 0.1 mg/ml anatase and 0.1 mg/ml rutile TiO_2_ for 24 h

In attempt to detect other changes in cell membrane after exposure to TiO_2_ for 24 h series of ion current measurements were performed as a function of voltage. As shown in Fig. 9 exposure to anatase has no effect on current density. In case of rutile only slight increase was observed in current density over 10–90 mV range.

**Fig. 10:**
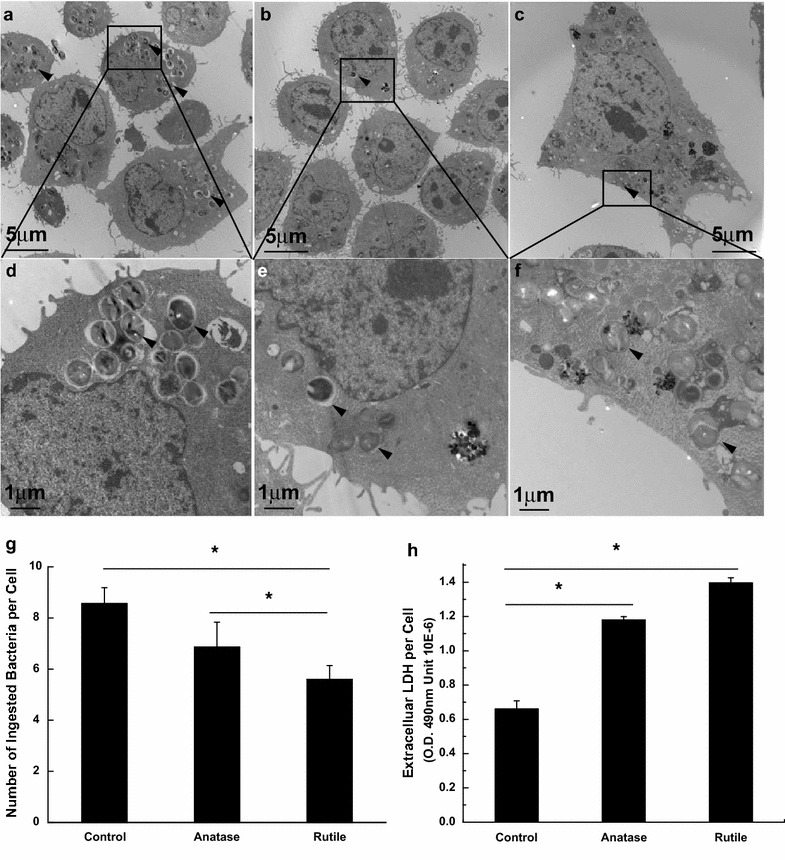
TEM cross sections of J77A4.1 control cells (**a**, **d**); cells exposed to 0.1 mg/ml anatase (**b**, **e**), and to 0.1 mg/ml rutile TiO_2_ (**c**, **f**) followed by exposure to *S. aureus* bacteria for 90 min. *Arrows* point to bacteria. **g** Number of ingested *S. aureus* bacteria per J77A4.1 macrophage cell exposed to anatase, rutile and unexposed to TiO_2_ nanoparticles. **h** Extracellular LDH levels in J77A4.1 cells exposed to TiO_2_ NPs and control unexposed cells. *Means P < 0.05

To further illuminate the effect of TiO_2_ exposure on bacterial infection we check the ability of macrophages to clear bacteria from the environment. The results on Fig. 10a–c show that exposure to TiO_2_ NPs for 24 h didn’t induce morphological changes in J77A4.1 macrophage cells. However, approximately 40 % fewer bacteria were ingested by J77A4.1 macrophages exposed to 0.1 mg/ml TiO_2_ NPs, as compared to unexposed control (Fig. 10d). Additionally, increase in extracellular LDH in J77A4.1 cells treated with TiO_2_ NPs was observed (Fig. 10e) and correlated reversely with bacteria uptake.

## Discussion

### Nanoparticle characterization

TiO_2_ NPs have been used progressively more in numerous commercial products, however there is an inadequate knowledge concerning effects of TiO_2_ nanoparticles on human health and environment. Although much research have reported that TiO_2_ NP exposure leads to ROS mediated cytotoxicity in various cell types [50–52], effects of TiO_2_ NPs not associated with the generation of ROS were not studied. In this manuscript we attempted to elucidate some fundamental aspects regarding the effects of TiO_2_ nanoparticles on cellular membrane mechanics and behavior. HeLa cells were chosen for the study due to their abundant use as a model system in anti-cancer research combining TiO_2_ nanoparticles and UV light. In addition to that, we wanted to be able to measure ion currents after treatment with nanoparticles and HeLa cells are perfectly suitable culture for patch clamping technique.

While the common approach in studying effects of TiO_2_ nanoparticles on cells is to measure cell proliferation decrease after exposure to high nanoparticles concentration [53] we decided to focus on the concentrations that do not affect cell proliferation. Our results allow a deeper understanding of cell membrane alterations induced by TiO_2_ nanoparticles, in the context of how changes in membrane mechanics can alter cell-bacteria interaction.

The change observed in particle surface charge after incubation in DMEM is expected and related to protein adsorption on nanoparticle surface. The medium used was supplemented with 10 % FBS with albumin being the dominant protein in it. It is well known that albumin is a main component of blood and the most prevailing protein in plasma [54] and it also has high affinity toward TiO_2_ particles under different experimental conditions [55, 56] especially in mixture with human fibrinogen [57, 58].

It was previously reported that negatively charged nanoparticles tend to adsorb proteins with isoelectric point greater than 5.5 [59]. According to Deng et al. [60] TiO_2_ nanoparticles mostly adsorb albumin and apolipoproteins from DMEM supplemented with FBS. In our experiment protein layer adsorbed on the surface altered nanoparticles’ net surface charge reducing it by 80 % in case of anatase and by 70 % in case of rutile nanoparticles. That is in agreement with observations by Allouni et al. [57] who reported TiO_2_ zeta potential change from −16 ± 2 in RPMI alone to −9 ± 1 mV in RPMI medium containing 10 % FBS.

Adsorption of proteins on surface of nanoparticles plays a critical role in particle stability. Several research groups [57, 61] shown that TiO_2_ agglomerates sediment rapidly in the absence of albumin, whereas in the presence of albumin nanoparticles formed stable suspension and sedimentation rate was close to zero over a 20-h period. Similarly, we observed prolonged TiO_2_ suspension stability in full DMEM compared to stability of water based suspensions.

The explanation of negatively charged albumin molecules spontaneously adsorbed on negatively charged TiO_2_ was proposed by Oliva et al. [55], who speculated that driving force of the adsorption may arise from structural modifications in the protein in contact with the oxide, and from interactions with chemicals that compensate the electrostatic repulsions.

### Nanoparticle interaction with cell

Neither the size of particles nor their surface charges had an influence upon intracellular particle sequestration. Inspection of the size distribution of aggregates showed a tendency for rutile TiO_2_ nanoparticles to have aggregates in a larger range (>500 nm), when compared to anatase. The mechanism of sequestration for both nanoparticles appears to be similar with particles being predominantly stored in vacuoles around the cytoplasm. Membrane-enclosed particle aggregates of different sizes were often observed next to nucleus but never inside of it. Major organelles including mitochondria, Golgi apparatus and rough ER were also found to be devoid of TiO_2_. As such, our present data are in agreement with previous observations about nano-sized TiO_2_ [62] and is in contrast with observations about ultrafine TiO_2_ particles that were found within mitochondrial membranes [63] and also inside nuclei [64]. The cellular uptake of TiO_2_ NPs was also confirmed by the flow cytometry. As Pan et al. [65] demonstrated, TiO_2_ NPs have a natural fluorescence, hence, the average intensity of the natural fluorescence can be correlated with the amount of particles taken up by the cell and used as a qualitative confirmation of the particle penetration. To validate this statement, we also performed flow cytometry measurement of cell granularity (Additional file 1: Figure S4). The increase in the side scatter intensity (SSC) and decrease in forward scatter intensity (FSC) were previously demonstrated to be related to the changes in the refractive index of cells containing TiO_2_ NPs [66]. Our data show distinct shifts in SSC and FSC proving TiO_2_ NPs uptake by the cells. These results are consistent with TEM micrographs demonstrating TiO_2_ NPs uptake by HeLa cells and indicate that uptake is slightly higher for anatase TiO_2_ NPs as compared to rutile.

Similar to many studies [62, 67, 68] we determined that nanoparticles penetrate cell membrane by means of endocytosis. It is known that bafilomycin A, a plecomacrolide antibiotic containing a 16-membered lactone ring, is a potent inhibitor of vacuolar ATPases which prevents acidification of endosomes [69] and thereby serves as potential inhibitor of NPs uptake [70, 71]. Our data reveals that pretreatment with bafilomycin A completely suppressed the uptake of TiO_2_ as compared to control cells thus indicating that uptake occurred through an active metabolism and confirming that TiO_2_ nanoparticles penetrate cells via endocytosis.

### Toxicity of nanoparticles

The concentration of TiO_2_ nanoparticles chosen for these experiments did not affect cell proliferation rates for 48 h and did not lead to significant increase in ROS. Therefore, the observed changes in HeLa cell behavior is not correlated with increased oxidation and cell death.

Lactate dehydrogenase (LDH) release test was used as an indicator of cell membrane porosity due to interactions with the TiO_2_ nanopaticles. It has been reported that LDH binds to TiO_2_ nanoparticles [72, 73], however in our experiments concentrations of rutile and anatase were kept below the concentrations at which differences in LDH readout could be detected.

We found that 24 h exposure to TiO_2_ rutile and anatase NPs at concentrations 0.1 mg/ml resulted in a significant extracellular LDH in the media. Total amount of intracellular LDH was found to be unchanged (although with a slight decrease in the cells treated with TiO_2_) proving that treatment with nanoparticles do not stimulate LDH production but induced membrane porosity. The increase in LDH release doesn’t correlate with proliferation pattern and suggests toxicity pathway other than apoptosis. These alterations in cell membrane were also accompanied by changes in cell morphology: we observed loss of cell junctions and impaired integrity of HeLa cells layer. Similar observation was done by Setyawati et al. [74], who reported that exposure to TiO_2_ nanoparticles lead to the disruption of cell–cell interactions in endothelial cells. Authors speculated that TiO_2_ nanoparticles are small enough to bind directly to VE–cadherin, resulting in the interruption of cell–cell connections, however in case of HeLa cells it seems to be unlikely because of its failure to express VE-cadherin.

### Nanoparticle-cell-bacteria interactions

The most intriguing results were obtained in experiment where HeLa cells were pretreated with TiO_2_ followed by exposure to *S. aureus* bacteria. Our data shows that number of bacteria associated with HeLa cell membrane increased in cultures pretreated with TiO_2_ nanoparticle. In addition, a significant increase in the number of bacteria per cell indicated that the cell membrane became more permeable to the bacteria. It is well established [75] that prior to infection pathogens first adhered to the cell through protein-carbohydrate interactions. This intervention strategy relies on adhesion that is driven by proteins on the pathogen that binds to carbohydrate structures displayed on the surface of cell. Moreover, similar to many human bacteria with oligosaccharide targets, *S. aureus* uses protein–carbohydrate recognition for adhesion [76]. Therefore, in an attempt to explain the increased number of bacteria associated with the cell membrane and also the increased number of bacteria per cell, we performed cell membrane staining for carbohydrates (data are not shown). However, that experiment did not reveal any significant difference between the cultures exposed to TiO_2_ and the control in the amount of membrane bound carbohydrates. Further, we investigated inhibition of bacteria adhesion to the cell membrane. The inhibitory effect of dextran on pathogens attachment was previously reported by Barghouthi et al. [77] where the effect was observed with several bacteria strains including *S. aureus*. The inhibition was identified as non-specific as dextran and other neutral polysaccharides didn’t bind neither to cell membrane nor to bacteria. Our results demonstrated that unlike control unexposed to TiO_2_ NPs, cultures exposed to rutile and anatase followed by dextran treatment exhibited decrease in bacteria uptake by 3.5 and fivefolds respectively. Such dramatic decrease in bacteria uptake suggests that TiO_2_ NPs promotes bacteria adhesion and thus uptake. As Jucker et al. reported [78] polysaccharides such as dextran have high affinity to TiO_2_ surface. Dextrans of various molecular weights were demonstrated to establish hydrogen bonds with hydroxyl groups on TiO_2_ surface or interact with surface bound water leading to irreversible absorption. It is also important to note, that treatment with dextran didn’t change TiO_2_ NPs uptake by the cells (Additional file 1: Figure S4). Therefore, we speculate that TiO_2_ NPs bound/incorporated into cell membrane adsorb bacterial polysaccharides increasing rates of bacterial attachment to the HeLa cell membrane and therefore increasing uptake of bacteria.

We also would like to mention that increased bacteria uptake can be related to increased LDH traffic through membrane. The cells secrete ROS and reactive nitrogen species (RNS) that are highly toxic to pathogens and are used to prevent tissue colonization by microorganisms [79, 80]. On the other hand, a wide variety of bacteria can inhibit ROS and RNS production in host cells and thus increase the possibility of persistent infection by promoting microbial survival within the host cell environment [79, 81]. Specifically, *S. aureus* can evade numerous components of host innate immunity [42, 44], including antimicrobial radical nitric oxide, by expressing an l-lactate dehydrogenase [79]. All the factors mentioned above can allow *S. aureus* to sustain redox homeostasis during nitrosative stress and stay virulent. Therefore, it is possible to speculate that increased LDH traffic through cell membrane might be recognized by *S. aureus* as a “safe” environment and attract larger number of bacteria toward the cell membrane.

The increased membrane stiffness in cultures treated with anatase can be explained by anatase having higher number of hydroxyl groups (-OH) on its surface compared to rutile [82]. Hydroxyl groups are known to lead to higher binding of nanoparticles to the cell membrane thereby increasing cellular membrane rigidity [14]. Similarly, Santos da Rosa et al. [83] observed increased stiffness of the membrane in neutrophils treated with TiO_2_ nanoparticles. It’s known that negatively charged TiO_2_ particles bind preferentially to amino acids with -OH, -NH, and -NH_2_ in their side chains [84]. Thus, TiO_2_ NPs possibly can impair cell membrane function by reacting with cell membrane proteins and leading to protein aggregation/denaturation [14].

The patch clamping experiment did not reveal any statistically significant difference between cultures treated with TiO_2_ and control. The increased fluctuation in ion currents in cultures treated with nanoparticles suggests difference in intracellular concentrations of nanoparticle. Similarly, Shah et al. [85] reported no changes in calcium and potassium channel activity of enteroendocrine cells treated with polymeric nanoparticles. On the other hand, our data is in contradiction with the findings of Chen et al. [86] and Busse et al. [87] who reported an increase in the ion currents in cultures treated with different nanoparticulate materials. It is interesting to note that in our experiments only 10–20 % of cells in cultures pretreated with TiO_2_ nanoparticles were successfully patched, the rest of cells in the culture failed to form a seal and eventually ruptured. That might be explained by the fact that cells accumulating nanoparticles exhibited more rigid membranes and are more prone to breakage under external force. In contrast, more than 95 % of control cells were easily patched from the first attempt. Thus, we speculate that patch clamping data presented here, and possibly in other publications, are exclusion bias due to the inability to patch cells that have higher amount of extracellular and membrane associated nanoparticles.

It is interesting to note that the TiO_2_ particles have an opposite effect on the interaction of *S. aureus* bacteria with J77A4.1 macrophages. In this case, number of bacteria ingested by macrophage cells didn’t increase. Internalization is a primary function of macrophages, which occurs by endocytosis followed by sequestration of bacteria and their destruction inside cell. Here we observed that fewer bacteria were ingested by macrophages exposed to TiO_2_ NPs. It is not clear if this phenomenon is related to the presence of particles which compete for space with bacteria inside of cytoplasm, versus impairment of cell membrane and endocytosis process. In any case, these results are consistent with previous observations of reduced bacteria clearance by macrophages pre-exposed to nanoparticles [88–90] in both murine and human models. It is also important to note, that several research groups recently reported compromised immune response in subjects pretreated with TiO_2_ NPs [91–93]. For example, Hong et al. [91] reported induction of reproductive toxicity and immunological dysfunction in male mice exposed to TiO_2_ NPs. Another researchers [92] showed that TiO_2_ NPs is immunotoxic to fish and reduces the bactericidal function of fish neutrophils. More detailed, fish exposed to TiO_2_ NPs had impaired host defenses to bacteria pathogens due to interactions of TiO_2_ NPs with innate immune cells and their progenitors. Such interactions significantly increased mortality and morbidity of the fish and also affected functioning of internal organs. In addition, Bechker et al. [93] reported pro-inflammatory effects of TiO_2_ NPs in human peripheral blood monocytes. It was shown that two biochemical processes closely related to the pathogenic infections are impaired: neopterin production is upregulated and the breakdown of tryptophan is suppressed. Authors note, that the consequences of these changes might lead to decreased immune response to the infection diseases. These findings suggest that the exposure to TiO_2_ NPs leads to multiple events that target immune system and compromise its bactericidal ability. Similarly, our results indicate that exposure of tissue to TiO_2_ nanoparticles may significantly increase the risk of bacterial infection. On one hand more bacteria are attracted in the vicinity of cells, while the macrophages are prevented from effectively removing the bacteria. The cells studied here were only model systems, but these results indicate that further studies should be performed on relevant models such as oral cavity or skin, where contact of epithelium or epidermis with TiO_2_ containing products and bacteria is common.

## Conclusions

We have found that exposure of HeLa cells to low concentrations of TiO_2_ nanoparticles may significantly increase the risk of bacterial invasion. HeLa cells cultured with 0.1 mg/ml rutile and anatase TiO_2_ nanoparticles for 24 h prior to exposure to bacteria had 350 and 250 % respectively more bacteria per cell, which might lead to further infection. The increase was associated with TiO_2_ NPs absorption of bacterial polysaccharides and an increase in extracellular LDH. In contrast macrophages exposed to TiO_2_ particles ingested 40 % fewer bacteria, further increasing the risk of infection. These concentrations of TiO_2_ did not affect cell proliferation or induce ROS generation. However it resulted in embrittlement of cell membranes and reduction in cell–cell connections.

## Methods

Anatase and rutile TiO_2_ nanopartilces were generously provided by cosmetic company. Trypsin–EDTA (0.05 %) (Catalog No: 25300-054) was purchased from Life Technology. Dulbecco’s Phosphate-Buffered Saline (Catalog number: 14190-250) was order from Life Technology.

### Cell plating

HeLa parental cells (ATCC CCL-2) were cultured in low glucose Dulbecco’s Modified Eagle Medium (DMEM) supplemented with 10 % Fetal Bovine Serum (FBS), and 1 % Penicillin–Streptomycin solution at 37 °C and 5 % CO_2_. For the experiments, cells were platted at average density of 20,000 cells per 35 mm^2^ Petri dish. After 24 h, TiO_2_ particles were added to the cell culture to obtain final concentration of 0.1 mg/ml.

J77A4.1 (ATCC TIB-67) cells were grown in the same conditions as stated above. Initial plating density and exposure to TiO_2_ was identical to the experiment with HeLa cells.

### Cell proliferation

To determine cell proliferation, cultures were plated at an initial density of 7500 cells per well in 12-well tissue culture plate and counted using hemocytometer at days 1, 2, and 3. Each grid square of the hemocytometer slide represents a volume of 10^−7^ m^3^, and cells were counted in 10 squares in 1 µl of the cell suspension. Each condition had triplicates and all experiments were conducted three times. Cell suspensions were mixed for uniform distribution and were diluted enough to prevent cell aggregation.

### Zeta potential

To prepare the samples, 2 µg of TiO_2_ NPs were put in 10 mL of deionized water or culture medium and sonicated for 5 min to separate agglomerates. After that samples were diluted 10 times, briefly sonicated and analyzed in a Brookhaven Instruments Zeta Plus Zeta Potential Analyzer. The average of three measurements of 50 cycles was used as a numerical value of zeta potential.

### Particles size and aggregation

Particle size measurements were performed using a BIC 90Plus dynamic light scattering (DLS) instrument (Brookhaven Instruments, Zeta Plus Zeta Potential Analyzer). To prepare the samples, 2 µg of TiO_2_ NPs were put in 10 mL of full culture medium for 24 h before analysis. After that samples were diluted 10 times with media and measured. The average of three measurements of 50 cycles was used as a numerical value of particle effective diameter.

### Patch clamping

Sutter Instrument Co. model P-97 flaming/Brown micropipette puller was used to convert 1.5 mm by 886 mm, 4” borosilicate glass capillary filament tubes (A-M Systems, cat #603000) into micropipettes. Before the patch clamping experiment began, a beaker of Tyrode’s bathing solution (Isotonic to the tissue culture) was connected via tube to the stage in order to moisturize the cells. Tweezers were used to lift the cover and place the slide on the stage of an inverted microscope (Olympus IMT-2). An individual cell, appearing healthy and not coated in NPs was then located using the microscope. Pipettes filled halfway with potassium-aspartate solution (135 µM) were used to facilitate current. The pipette was then attached to an electrode immersed in the solution. The electrode connected to an amplifier (Axon Instruments, AxoPatch 200B), acted as a current-to-voltage converter, and data acquisition system. The pipette was gently lowered to the cell using hydraulic manipulators. Once the tip of the pipette touched cell surface, resistance increased on the Axon instruments oscilloscope and positive pressure was released. Suction was applied to the patch pipette interior and formed a high resistance seal (a Gigaseal in the Giga Ohms range). After forming a gigaseal, the pipette applied suction to slightly rupture the membrane, and since the pipette was in contact with the cells interior, the electrode in the pipette measured and recorded the total cell patch current carried by flowing ions on the digital storage oscilloscope. Suction was applied at a gentle pace until large spikes appeared at the beginning and the end of test pulse. Following this, cell capacitance was recorded. The entire patch clamp apparatus rested on an anti-vibration table, within a Faraday cage to minimize electromagnetic disturbances.

### Fow cytometry

Cell were plated with starting density 0.2 million for 24 h and followed with 0.1 mg/ml TiO_2_ nanoparticles incubation for another 24 h. Both the control and the experimental cells were carefully rinsed more than three times to remove all the floating particles in the experimental media and detached with trypsin-ethylenediaminetetraacetic acid (EDTA). After stopping trypsin with full-DMEM, the cells were rinsed twice using DMEM with BSA (0.2 %) for good separation. Then cells were re-suspended in PBS at the concentration of 10^6^ cells/mL and sent for flow cytometry, which was performed with a BD FACSCaliburTM benchtop flow cytometer.

### Cell staining for confocal microscopy

Cell area and overall morphology as a function of nanoparticle uptake were monitored using a Leica confocal microscope. For these experiments, cells were exposed to TiO_2_ for 24 h as previously described and afterwards fixed with 3.7 % formaldehyde for 15 min. Alexa Fluor 488-Phalloidin was used for actin fiber staining and propidium iodide for nuclei staining.

### Lactate dehydrogenase activity (LDH) measurements

LDH was measured in cell culture media overlying the cells (extracellular LDH) and in the cell homogenates (intracellular LDH).

### Extracellular LDH measurements

Pierce LDH Cytotoxicity Assay Kit (#88953, Life Technology) was used for the experiment. After 24 h incubation with nanoparticles, 50 µl supernatant from each sample were transferred to a 96-well plate in triplicate wells and 50 µl of reaction mixture (lyophilizate mixture) were added. After incubation at room temperature for 30 min, reaction was stopped by adding 50 µl stop solution. Released LDH absorbance was measured at 490 and 630 nm respectively.

### Intracellular LDH measurement

Lactate Dehydrogenase Activity Assay Kit (MAK066, Sigma-Aldrich) was performed to examine cell LDH. To get optimum result, 10, 25, 50 K cells were added in triplicates to 96-well plate according to manufacturer’s instruction and LDH absorbance was read at 450 nm at 5, 10, 20, and 30 min respectively. Finally 50 K cells with reading at 10 min that exhibited best result was used to calculate LDH concentration (nM/50 K).

### Reactive oxygen species (ROS) quantitative measurement

Reactive oxygen species detection reagents (cat C6827, invitrogen) was used to detect ROS level of HeLa cells. For this experiment 50 µg 5-(and-6)-chloromethyl-2′,7′-dichlorodihydrofluorescein diacetate, acetyl ester (CM-H_2_DCFDA) was dissolved in 100 µl ethanol to make a stock solution. After that 100 µl of stock solution was diluted in 10 ml DPBS to make working solution. Cultures were grown and exposed to TiO_2_ for 24 h in 96-well dish. Then 100 µl of working solution was added to each well and incubated for 20 min. After that 100 µl of 20 mM NaN_3_ was added to each well and incubated for 2 h. Fluorescence was read at 490 nm excitation and 520 nm emission.

### Preparation of the bacteria

The *S*. *aureus* (ACTT25923) was obtained from ATCC. *S*. *aureus* was grown overnight at 37 °C with shaking at 200 RPM in modified brain heart infusion broth supplemented with 0.5 % yeast extract. The following day, the culture was diluted 1: 200 into fresh, pre-warmed broth until the culture reached mid-logarithmic phase (approximately an optical density of 2.0 at 600 nm). *S*. *aureus* cells were harvested by centrifugation at 1300×*g* for 10 min and the supernatant was discarded. The pellet was re-suspended in phosphate buffered saline (PBS) (pH = 7.2), again centrifuged and re-suspended in PBS to a density of approximately 1 × 10^10^ cells per ml.

### Bacteria infection

For cell infection experiment, the *S. aureus* was co-cultured with HeLa cells (or J77A4.1) for 90 min at 37 °C at the ratio 1:1000. After infection, all samples were further treated for characterization.

### Bacteria enumeration

*Staphylococcus aureus* were co-cultured with cells pretreated with nanoparticles and control at 1:1000 ratio for 90 min at 37 °C. Samples were washed three times with 1 ml of PBS each time with vigorous shaking to remove all non-adherent bacteria. Finally, tissue culture cells were lysed with 0.2 % Triton X-100 and intracellular bacteria were enumerated by serial dilution and plate counting.

After lysis of HeLa cells (or J77A4.1), colony forming units of *S. aureus* were determined by plating a series of 10-fold dilutions (in PBS), on blood agar plates and overnight incubation at 37 °C. All dilutions were plated in triplicate and the average ± standard deviation reported. Blood agar plates were composed of trypticase soy agar supplemented with 5 % defibrinated sheep blood. The number of bacteria per cell was obtained to divide bacteria number from colony counting by cell number.

### Dextran and TiO_2_ nanoparticle treatment

After incubation with Dextran (Sigma-Aldrich, 31,390) 1 mg/ml for 24 h, cells were incubated with 0.1 mg/ml nanoparticles again for another 24 h. Bacteria were added in at 1:1000 ratio and followed by 90 min infection; cell and bacteria were collected and sent for colony enumeration.

### Fluorescence microscopy

FilmTracer™ LIVE/DEAD Biofilm^®^ viability kit (Invitrogen, Cat. L7012) was used for bacteria staining After 90 min incubation with *S. aureus*, samples were stained by mixture of SYTO^®^ 9 and Propidium Iodide with 1 µl/mL according to manufacturer’s’ instruction in the dark for 15 min. After that samples were washed thoroughly three times with PBS to get rid of bacteria residue and observed immediately under Confocal microscope.

### Statistical analysis

All experiments were performed in triplicates and repeated at least three times. The results were represented as mean ± SD. A p value less than 0.05 was considered statistically significant.

### Bafilomycin treatment

Cells were treated with 100 nM bafilomycin A1 (Sigma-Aldrich) in 1 % DMSO for 1 h, then exposed to TiO_2_ for 4 h followed by fixation in mixture of 2.5 % of paraformaldehyde and 2.5 % glutaraldehyde in PBS and stained for TEM.

### Optical tweezers

**Fig. 11:**
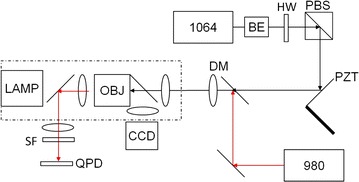
Schematic diagram of the experimental setup. *BE* beam expander; *HW* half-wave plate; *PBS* polarizing beam splitter; *PZT mirror* mirror mounted on a PZT piezo-electric; *DM* dichroic mirror; *OBJ* microscopic objective; *LF* 1000 nm short-pass filter; *QPD* quadrant photodiode

Optical trapping was achieved by an IR (1064 nm) laser coupled into an oil-immersion objective lens (100X, NA = 1.3, Olympus). A second laser beam (980 nm) aligned and focused by the same objective lens to be parfocal with the trapping laser focus was used for particle tracking. The 1064 nm laser is expanded and collimated to just overfill the back aperture of the objective lens, ensuring that a diffraction limited spot is created for particle trapping. To prevent contribution to the optical trapping effect, the 980 nm tracking beam power is attenuated to two orders of magnitude lower than that of the 1064 nm laser. Movements of the particle tracked by the 980 nm laser beam were detected by a quadrant photodiode (QPD). The voltage reading of the QPD was maintained on a linear function of the particle displacement from the trapping center. The wide-field images of the particle were captured by a CCD camera. This experiment setup is shown below in Fig. 11.

## Additional file

10.1186/s12951-016-0184-y Supplementary Data.
