# Supplementary material for: Exposure to TiO2 nanoparticles increases Staphylococcus aureus infection of HeLa cells
Source: J Nanobiotechnology. 2016 Apr 22;14:34. doi: 10.1186/s12951-016-0184-y (PMC4840899; doi:10.1186/s12951-016-0184-y)
Supplement: Supplementary file 1 — 10.1186/s12951-016-0184-y Supplementary Data. [file 12951_2016_184_MOESM1_ESM.pdf]

Supplementary Data

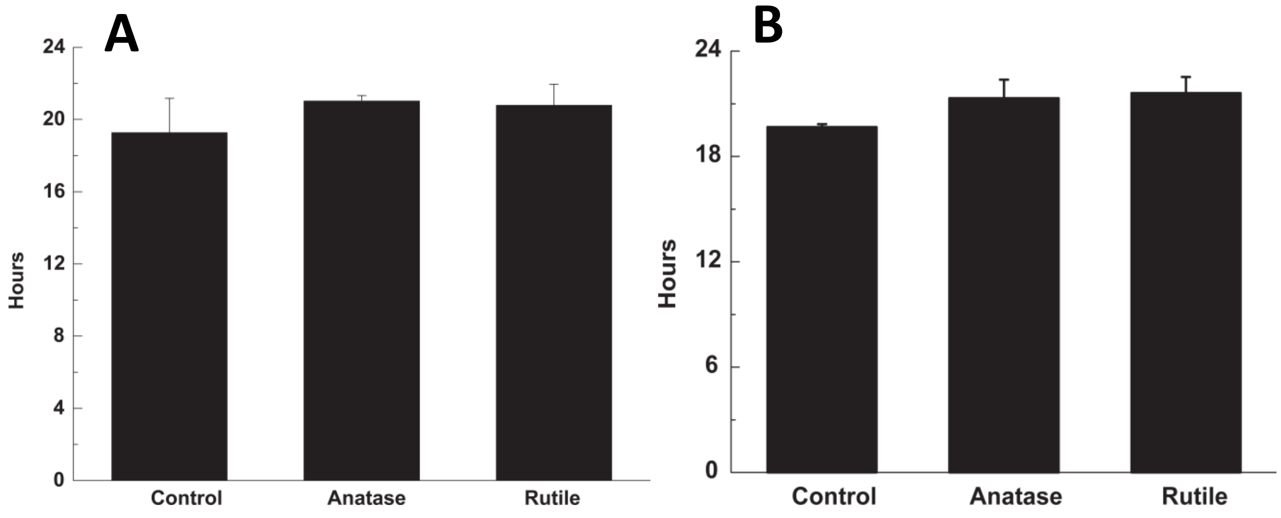

Figure 1. Cell population doubling times. A) After 2 days of exposure to TiO_2 NPs; B) After 3 days of exposure to TiO_2 NPs.

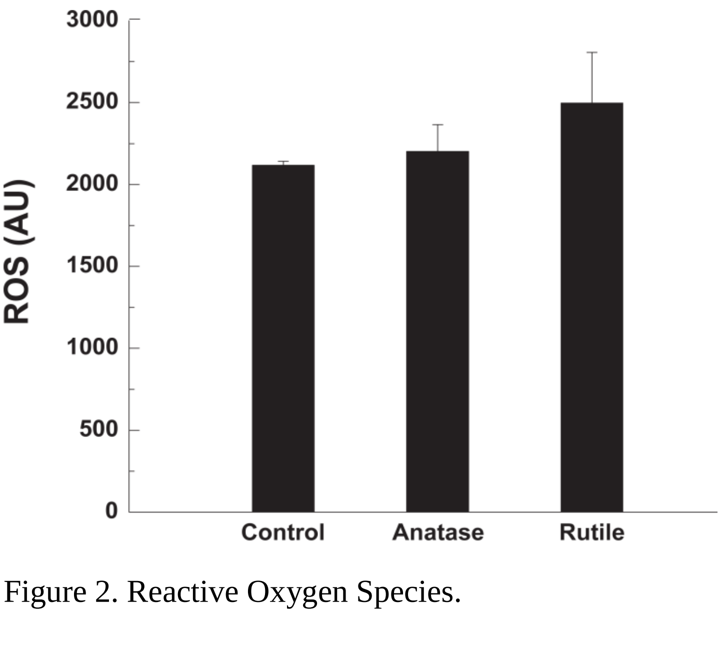

Figure 2. Reactive Oxygen Species.

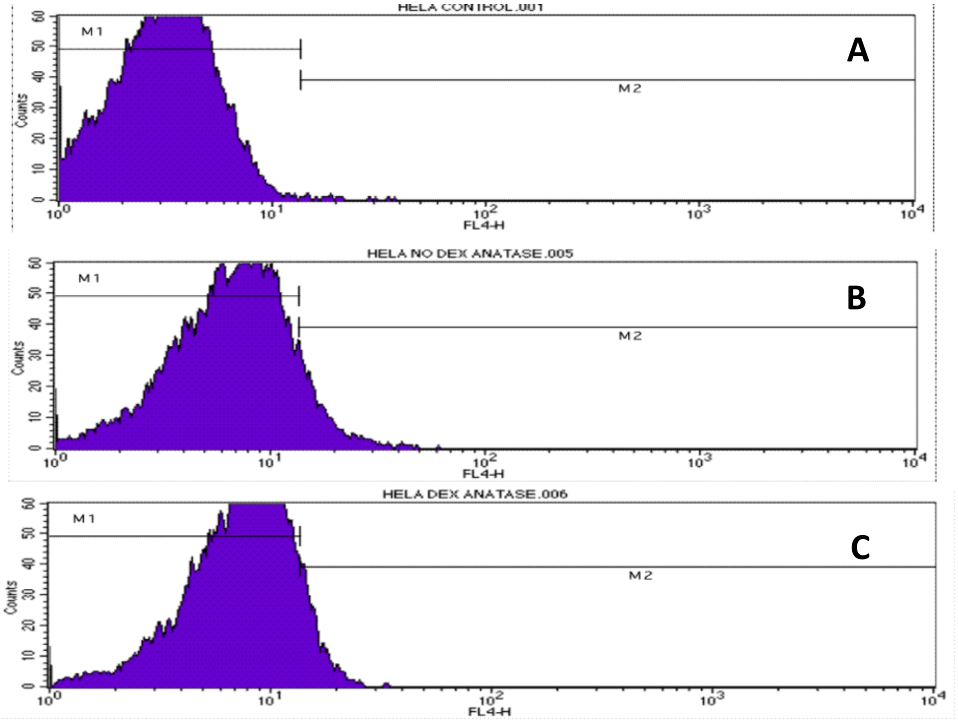

Figure 3. Cell population in fluorescent channel FL4-H gated with M1 marker (autofluorescence) and M2 marker (TiO_2 fluorescence). A) Control cells; B) Cells treated with anatase; C) cells treated with rutile.

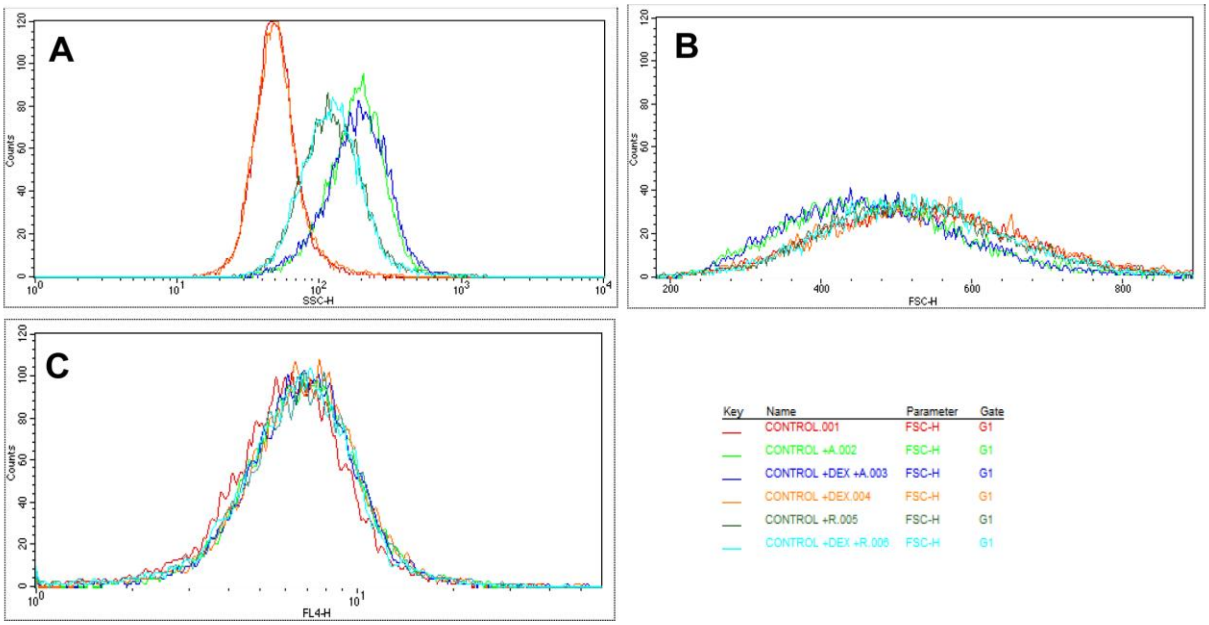

Figure 4. Shift in side scatter (SSC) intensity (A); forward scatter (FSC) intensity (B); and fluorescence (FLH-4)intensity (C) in control cultures (red), control cultures treated with dextran (orange); cultures treated with anatase (green); cultures treated with dextran and anatase (blue); cultures treated with rutile (black); cultures treated with dextran and rutile (sky blue).
